# Clinical, biochemical, and genetic analysis of two Korean patients with Trichorhinophalangeal syndrome type I and growth hormone deficiency

**DOI:** 10.1186/1687-9856-2013-S1-P59

**Published:** 2013-10-03

**Authors:** Young Bae Sohn, Chang-Seok Ki, Sung Won Park, Sung-Yoon Cho, Ah-Ra Ko, Min-Jung Kwon, Ji-Youn Kim, Hyung-Doo Park, Ok-Hwa Kim, Dong-Kyu Jin

**Affiliations:** 1 Department of Medical Genetics, Ajou University Hospital, Suwon, Korea; 2 Department of Laboratory Medicine and Genetics, Sungkyunkwan University School of Medicine, Seoul, Korea; 3 Department of Pediatrics Samsung Medical Center, Sungkyunkwan University School of Medicine, Seoul, Korea; 4 Center for Clinical Research, Samsung Biomedical Research Institute, Seoul, Korea; 5 Department of Radiology, Ajou University Hospital, Suwon, Korea

## 

Tricho-rhino-phalangeal syndrome type I (TRPSI) is a rare autosomal dominant hereditary disorder characterized by sparse hair, bulbous nose, long philtrum, thin upper lip, and skeletal abnormalities including cone-shaped epiphyses, shortening of the phalanges, and short stature. TRPSI is caused by mutations in the *TRPS1* gene. Herein, we report two Korean cases of TRPSI. Although both patients (a 17-year-old-female and a 14-year-old male) had typical clinical findings, Patient 1 had an additional growth hormone (GH) deficiency. Treatment with recombinant human growth hormone (rhGH) 0.7 IU/kg/week led to an increase in growth velocity. Over 10 years of GH therapy, the mean growth velocity was 5.7±0.9 cm/year. While patient 2 showed a low response after the GH stimulation test, the patient had a poor response with rhGH therapy and GH therapy was discontinued after 6 months.

For the genetic analysis of the *TRPS1* gene, two mutations were found. Patient 1 had a heterozygous mutation c.2520dupT (p.Arg841LysfsX3) which had not been previously reported. Patient 2 had a known nonsense mutation c.1630C>T (p.Arg544X). In summary, we were the first to report Korean patients with mutation of *TRPS1*.

